# The Expanding Role of Artificial Intelligence in Dentistry: A Cross-Specialty Chairside Perspective

**DOI:** 10.7759/cureus.98449

**Published:** 2025-12-04

**Authors:** Fahad S Albuhayri, Saad J Albshaier, Almiqdad I Dashti, Joud F Alrajhi, Fajer K Alhamidy, Mahdi A Busuhail, Fahad N Bujbarah, Mustafa K Rizq, Nada A Thubab, Safeyah A Takronni, Jihan I Alharbi, Aeshah H Hakami, Hadeel S Aloufi, Mohammed I Mathar

**Affiliations:** 1 College of Dentistry, Qassim University, Buraydah, SAU; 2 College of Dentistry, King Faisal University, Al-Ahsa, SAU; 3 Dental Administration, Ministry of Health, Kuwait City, KWT; 4 College of Dentistry, King Saud Bin Abdulaziz University for Health Sciences, Riyadh, SAU; 5 College of Dentistry, Imam Abdulrahman Bin Faisal University, Dammam, SAU; 6 College of Dentistry, Taibah University, Al-Madinah, SAU; 7 Faculty of Dentistry, King Abdulaziz University, Jeddah, SAU; 8 Faculty of Dentistry, Umm Al-Qura University, Makkah, SAU; 9 College of Dentistry, Jazan University, Jazan, SAU; 10 College of Dentistry, Mustaqbal University, Buraydah, SAU; 11 Prosthetic Dental Sciences, College of Dentistry, Qassim University, Buraydah, SAU

**Keywords:** ai, artificial intelligence, deep learning, digital dentistry, machine learning, teledentistry

## Abstract

Artificial intelligence (AI) has emerged as a transformative tool in healthcare, with dentistry increasingly integrating AI technologies to enhance diagnostics, treatment planning, patient monitoring, and clinical outcomes. The dental field, with its strong reliance on imaging, precision, and personalized care, offers fertile ground for AI-driven innovation. This narrative review aims to provide a comprehensive overview of current and emerging applications of AI across all major dental specialties. It explores the integration of AI technologies within clinical, educational, and administrative contexts. A literature search was conducted for English-language articles. The review synthesizes findings related to AI applications across dental specialties. AI has the potential to reshape dental care by enhancing precision, efficiency, and accessibility. Continued research, interdisciplinary collaboration, and ethical implementation are essential to ensure that AI serves as a supportive, equitable, and reliable force in modern dentistry.

## Introduction and background

Artificial intelligence (AI) has emerged as a transformative force across numerous sectors, and its integration into healthcare is rapidly reshaping traditional clinical paradigms. Within dentistry, AI holds significant promise for enhancing diagnostic precision, streamlining clinical workflows, personalizing patient care, and augmenting treatment outcomes. As digital technologies become increasingly central to dental practice, AI is evolving from a theoretical concept into a tangible asset in both research and clinical settings [1]. The roots of AI date back to the mid-20th century, with foundational work in computer science, neural networks, and algorithmic decision-making. Over the past two decades, exponential growth in computational power, data availability, and algorithmic sophistication has enabled AI systems to outperform conventional tools in tasks such as pattern recognition, classification, and predictive analytics. In dentistry, these capabilities are particularly relevant, given the field’s reliance on high-resolution imaging, precise morphological analysis, and complex clinical decision-making processes [2].

AI includes a wide spectrum of technologies, including machine learning (ML), deep learning (DL), natural language processing (NLP), and computer vision [3]. These systems are designed to simulate human cognitive functions such as learning, reasoning, and problem-solving. In the dental context, AI algorithms have demonstrated efficacy in detecting carious lesions, classifying periodontal status, segmenting root canals, predicting orthodontic outcomes, and optimizing prosthetic designs [4]. Beyond its diagnostic capabilities, AI is also impacting dental education, practice management, and patient communication. Intelligent tutoring systems, virtual simulation platforms, and predictive scheduling tools exemplify how AI can support clinicians and students alike [5]. Moreover, AI-driven tools are becoming increasingly relevant in teledentistry, offering support for remote consultations and triage, especially in underserved populations [6]. The rapid integration of AI into dental practice raises both opportunities and challenges. On one hand, these technologies can significantly enhance clinical efficiency and precision. On the other hand, concerns about data privacy, algorithmic transparency, ethical accountability, and regulatory oversight require careful consideration. As AI systems continue to evolve, the dental profession must adapt to ensure responsible and evidence-based implementation.

Therefore, this narrative review aims to provide a comprehensive overview of the current state of AI in dentistry, highlighting its applications across all major dental specialties. It also explores the underlying technologies, clinical impacts, ethical considerations, and future directions that will shape the role of AI in oral healthcare. By synthesizing recent developments and expert insights, this review serves as a foundational resource for researchers, clinicians, and policymakers seeking to navigate the intersection of AI and modern dental practice.

## Review

Search strategy

A comprehensive literature search was conducted to identify relevant studies addressing the application of AI in dentistry. The search was designed to capture a wide range of AI technologies and their clinical, diagnostic, educational, and operational applications in dental practice. The databases PubMed (MEDLINE), Scopus, Web of Science, and ScienceDirect were searched. The search was restricted to publications in the English language. A combination of Medical Subject Headings (MeSH) and free-text terms was used to ensure sensitivity and specificity. The following search string was adapted for each database: ("artificial intelligence" OR "machine learning" OR "deep learning" OR "neural networks" OR "computer vision" OR "natural language processing") AND ("dentistry" OR "dental" OR "oral health" OR "oral surgery" OR "orthodontics" OR "restorative dentistry" OR "endodontics" OR "periodontology" OR "pediatric dentistry" OR "oral pathology" OR "oral medicine" OR "dental radiology" OR "teledentistry"). Boolean operators and database-specific filters were used to refine the results. Inclusion criteria included articles discussing the development, application, or evaluation of AI technologies in any dental specialty, studies covering diagnostic tools, treatment planning, clinical decision support, teledentistry, robotics, or educational applications, and articles published in English between January 2018 and March 2025. Exclusion criteria comprised articles unrelated to dentistry or oral health, non-English publications, and conference abstracts, commentaries, letters, and articles lacking substantial data or methodological reporting. The selected literature formed the basis of a thematic synthesis structured around the major branches of dentistry and cross-cutting issues related to AI implementation.

Artificial intelligence technologies and methodologies in dentistry

AI encompasses a broad range of computational systems designed to mimic human cognitive abilities, including learning from data, recognizing patterns, and making autonomous decisions. The integration of AI into dentistry relies on several core subfields, primarily ML, DL, and NLP. Each plays a distinct role in enabling computers to interpret clinical data and perform tasks that traditionally require human expertise [7].

Machine Learning

At the heart of many dental AI applications lies ML, a technique that enables systems to improve their performance over time without being explicitly programmed. ML algorithms are trained using structured datasets to perform predictive tasks, such as classifying radiographic findings or estimating treatment outcomes. Supervised learning is the most commonly used ML approach in dentistry, with applications that include identifying types of dental lesions, predicting caries risk, and classifying periodontal disease severity [8].

Deep Learning

**Figure 1:**
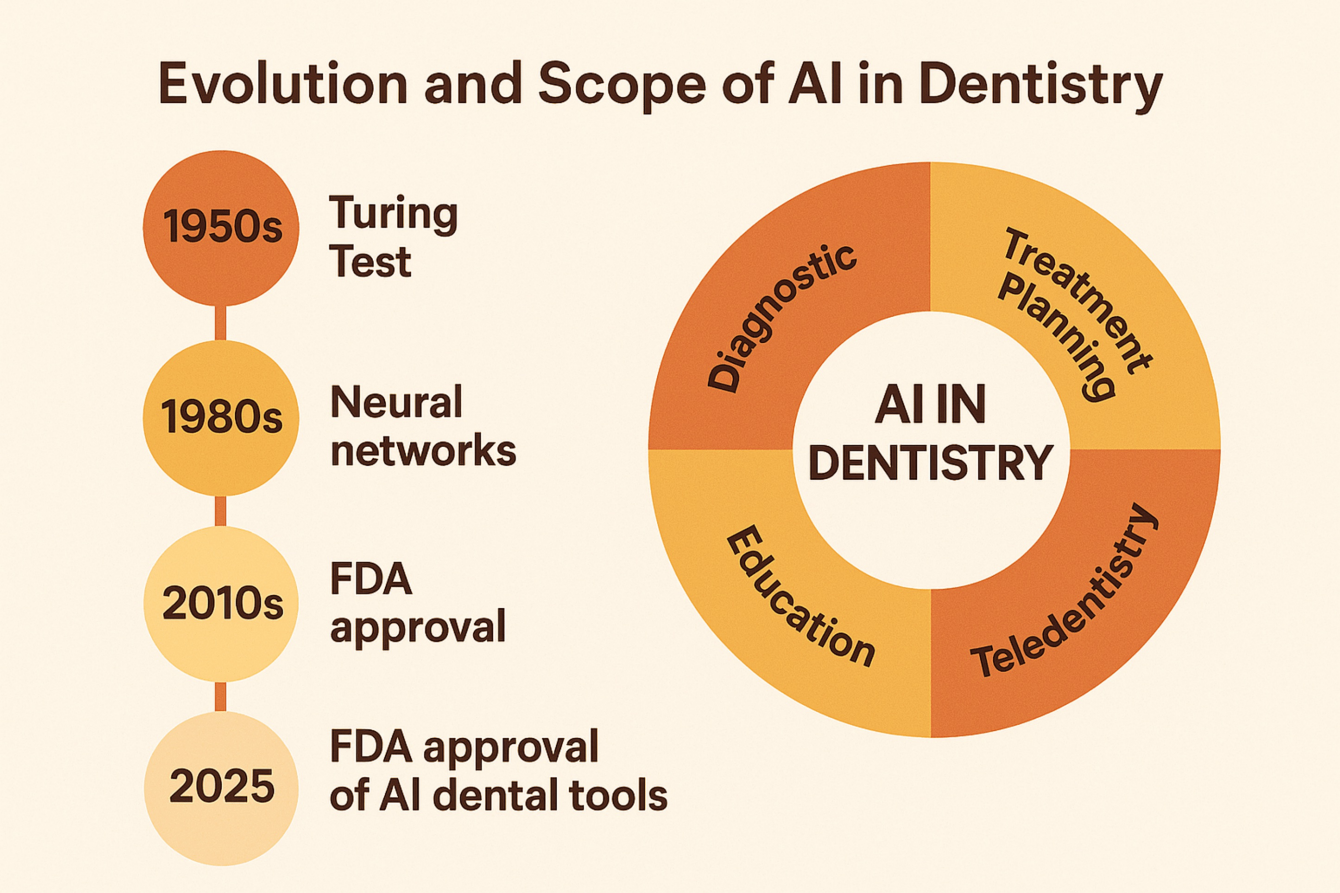
Historical milestones and clinical applications of artificial intelligence in dentistry Image credits: Authors

A subset of ML, DL leverages multilayered artificial neural networks that can analyze complex patterns in high-dimensional data, such as images. The most widely applied DL model in dentistry is the convolutional neural network (CNN), which has demonstrated remarkable success in interpreting dental radiographs. These models automatically extract and learn relevant features from the input data, making them particularly suitable for tasks like segmentation of anatomical structures, lesion detection, and landmark identification [9]. As illustrated in Figure 1, the integration of AI into dentistry has evolved over several decades, with applications now spanning diagnostics, treatment planning, robotics, teledentistry, and education.

Natural Language Processing

NLP enables AI systems to interpret, analyze, and generate human language. Although its adoption in dentistry is still emerging, NLP has potential applications in extracting useful clinical insights from unstructured text in electronic health records (EHRs), automating charting, and enhancing patient communication through intelligent chatbots and virtual assistants [10].

Reinforcement Learning and Emerging Models

Although less commonly applied in dentistry, reinforcement learning may be useful in robotic surgical systems and complex treatment simulations. Additionally, transformer models widely used in general AI are now being explored for dental education and diagnostic dialogue systems [11].

Data Sources and Integration

Effective AI implementation depends on high-quality input data. In dentistry, common data sources include 2D/3D imaging, intraoral scans and photos, EHRs, genomic and salivary biomarker data, wearable devices, and biosensors. The ability of AI systems to integrate and analyze multimodal data is increasingly valuable, offering the potential to support more holistic, personalized dental care [12].

Model Training and Validation

For AI models to be clinically reliable, they must undergo rigorous training and validation using large, diverse, and well-annotated datasets. Cross-validation techniques, test-train splits, and external validation are essential to assess model generalizability. Unfortunately, many published dental AI models remain limited by small datasets, lack of external validation, or overfitting, which impairs clinical applicability [13]. The primary AI technologies employed in dental applications, along with their core functions, are summarized in Table 1.

**Table 1:** Artificial intelligence technologies utilized in dental applications ML, machine learning; DL, deep learning; NLP, natural language processing; CNNs, convolutional neural networks

Technology	Definition	Common Applications in Dentistry
ML	Algorithms that learn patterns from data	Caries prediction, periodontal disease classification
DL	Subset of ML using neural networks with multiple layers	Radiograph interpretation, lesion detection
CNNs	Specialized for image recognition	Cephalometric analysis, panoramic X-ray diagnosis
NLP	Understanding and generating human language	Chart review, auto-generated reports, patient communication bots
Computer vision	Visual data processing and interpretation	Tooth segmentation, restoration design, intraoral imaging analysis
Reinforcement learning	Learning through trial-and-error with feedback	Robotics, AI-guided surgical maneuvers

Clinical applications of artificial intelligence across dental specialties

Orthodontics

Orthodontics has been at the forefront of AI integration within dentistry, largely due to the field's reliance on diagnostic imaging, longitudinal growth monitoring, and precision in treatment planning. AI applications in orthodontics range from diagnosis and classification of malocclusions to automated treatment planning and real-time monitoring [14]. A primary advancement is the use of DL for automated cephalometric analysis. CNNs trained on thousands of radiographs can now detect craniofacial landmarks with high accuracy. This provides clinicians with reliable measurements for skeletal and dental relationships. These tools reduce the time-consuming nature of manual tracing and improve consistency, which is particularly valuable in large-scale epidemiological studies and longitudinal treatment assessment [15]. AI is also integrated into clear aligner technologies, such as those used by commercial platforms like Invisalign. Algorithms help simulate tooth movements, design stepwise aligner stages, and forecast treatment duration. This automation enhances clinician efficiency and supports individualized treatment plans optimized for the patient's occlusal and skeletal features [16]. Another rapidly evolving application involves malocclusion detection and classification using intraoral scans and facial photographs. AI-based systems can categorize malocclusions according to Angle's classification or more complex criteria, assisting in early diagnosis and standardization. These models have been used to predict treatment need and generate comprehensive orthodontic records [17]. Digital tools often provide detailed diagnostic information, sometimes reducing the necessity for conventional radiographic imaging. This lowers the patient's exposure to radiation and streamlines diagnostic and treatment planning processes, enabling faster and potentially safer orthodontic care [18]. AI has also been used to develop predictive models of craniofacial growth, enabling clinicians to better time interventions during periods of rapid development. This is especially important in treating Class II or Class III malocclusions in growing patients. Accurate anticipation of growth spurts allows orthodontists to initiate treatment at the optimal stage of development, maximizing the potential for natural correction and possibly minimizing the need for invasive procedures [19]. ML models can analyze subtle patterns and changes in craniofacial structures that may be overlooked with traditional assessment methods, offering nuanced insights into each patient's unique growth trajectory [20].

AI is also transforming patient monitoring and compliance assessment. Applications that use smartphone cameras and AI-based analysis allow for remote progress tracking and flag issues such as poor aligner fit or insufficient tooth movement between appointments. These tools support teledentistry models, increase accessibility, and enhance patient adherence. This shift supports better treatment outcomes and promotes a more patient-centered approach to care, especially for those with limited access to in-person consultations [21]. AI in orthodontics enhances diagnostic capability, supports precise treatment planning, and enables more dynamic, remote, and patient-centered care models. As AI continues to evolve, its integration will likely expand into growth forecasting, occlusion modeling, and real-time chairside decision support [22].

Oral and Maxillofacial Surgery

AI has begun to revolutionize the field of oral and maxillofacial surgery (OMFS) through its integration in diagnostic imaging, surgical planning, intraoperative guidance, and postoperative outcome prediction. Given the complexity of craniofacial anatomy and the high precision required in surgical interventions, AI provides tools that enhance decision-making, safety, and efficiency [23]. One of the most established applications is in 3D imaging analysis. DL models, particularly CNNs, have been trained to interpret cone-beam computed tomography (CBCT) and computed tomography (CT) scans for tasks such as automatic segmentation of anatomical structures, detection of impacted teeth, identification of pathologies (e.g., cysts, tumors), and classification of facial fractures. These tools support clinicians in preoperative evaluations by reducing the time required for manual interpretation and minimizing interobserver variability [24].

In orthognathic surgery, AI is used to simulate osteotomies, forecast postoperative soft tissue adaptation, and generate virtual treatment plans. Some platforms integrate AI with 3D planning software to enable accurate surgical simulation and visualization of outcomes, improving both surgical precision and patient communication. Algorithms have also been trained to predict postoperative complications, including infection risk, delayed healing, or nerve injury, based on preoperative patient and procedural data [25]. AI plays an expanding role in implantology, where ML models predict implant success and bone integration by analyzing patient-specific parameters such as bone density, systemic conditions, and occlusal forces. Predictive analytics can also assist in determining the ideal implant site and angulation [26].

In robotic-assisted surgery, AI algorithms are being integrated into robotic platforms to allow for precision-controlled instrument movement, responsive adaptation to intraoperative variables, and minimally invasive procedures. Though still in its infancy in dentistry, AI-assisted robotics has shown promise in orthognathic procedures and implant placement. AI in OMFS improves diagnostic accuracy and surgical outcomes and supports a more personalized approach to complex interventions, aligning surgical planning more closely with individual patient anatomy and risks [27].

Restorative Dentistry

In restorative dentistry, AI applications are primarily focused on the early detection of carious lesions, evaluation of restoration margins, material selection, and treatment planning support. Given the discipline’s reliance on visual and tactile diagnostics, AI provides meaningful enhancements through high-resolution imaging analysis and real-time data interpretation [28]. One of the most impactful applications is in dental caries detection. DL algorithms trained on large datasets of bitewing and periapical radiographs have shown high accuracy in identifying incipient and cavitated lesions. These models can often match or exceed human performance, especially in detecting occlusal and approximal lesions, which are prone to underdiagnosis in routine practice. AI systems also support the standardization of diagnostic decisions, reducing variability between practitioners and improving patient care quality [29].

AI has also shown utility in assessing the integrity of restorations, including detecting marginal leakage, overhanging restorations, and secondary caries. By leveraging CNNs and other imaging-based classifiers, these systems can rapidly flag compromised restorations that require monitoring or intervention [30]. In material selection and treatment planning, AI tools can process patient-specific variables (e.g., occlusion, parafunctional habits, caries risk, and esthetic demands) to recommend suitable restorative materials and techniques. Some models have also been trained to predict the longevity of direct restorations based on clinical, behavioral, and environmental data [31]. Another important advancement is automated cavity classification. AI algorithms using intraoral cameras and radiographs can assign classes (e.g., Class I-VI) to carious lesions, guiding clinicians toward appropriate preparation techniques. These systems reduce the reliance on subjective judgment and are especially valuable in educational contexts [32].

In digital restorative workflows, AI facilitates automated segmentation and modeling during the design of onlays, inlays, and veneers. It can also assist in predicting post-restoration color match and esthetic blending, addressing one of the key challenges in anterior restorations. AI in restorative dentistry enhances early detection, reduces diagnostic errors, and contributes to individualized, data-driven restorative care. With continued advances, AI will likely become a standard adjunct to clinical judgment in the detection, monitoring, and planning of direct and indirect restorations [33].

Pediatric Dentistry

The application of AI in pediatric dentistry is gaining momentum, driven by the need for early diagnosis, behavior prediction, and preventive care strategies tailored to young patients. AI offers tools that enhance the accuracy of caries detection, streamline clinical decision-making, and improve communication with children and caregivers [34]. One of the most widely studied applications is the early identification of dental caries in primary teeth. DL models have shown exceptional performance in analyzing bitewing and periapical radiographs to detect incipient lesions. These systems help clinicians intervene earlier and reduce the reliance on invasive procedures. Compared to traditional visual-tactile assessment, AI-enhanced image analysis minimizes subjectivity and supports standardized diagnosis across providers [35]. AI has also been explored for predicting caries risk based on behavioral, dietary, and socioeconomic factors. ML models can integrate variables such as fluoride exposure, brushing habits, sugar intake, and access to dental care to forecast individual risk profiles. These tools assist in establishing customized preventive programs and recall intervals [36]. Beyond caries management, AI plays a role in behavioral prediction, particularly for anticipating treatment compliance and the need for behavior management techniques. By analyzing historical behavioral data, age, parental anxiety, and previous dental visits, AI models can help clinicians prepare for potentially challenging appointments and select appropriate behavior guidance strategies (e.g., tell-show-do, sedation) [7].

In growth monitoring, AI supports the longitudinal evaluation of dental and skeletal development through cephalometric landmark detection and analysis. This aids in identifying developmental disturbances and planning interceptive orthodontic treatments. Additionally, algorithms have been developed to predict tooth eruption timing, arch space availability, and the likelihood of crowding, all of which inform early intervention planning [37]. Educationally, AI is being incorporated into child-focused teledentistry platforms that use visual recognition and interactive modules to promote oral hygiene among children. These technologies support engagement, particularly in underserved or remote populations where access to regular dental care is limited. AI in pediatric dentistry facilitates accurate, early, and preventive care while also supporting behavioral and developmental management. Its ability to integrate clinical, behavioral, and social data aligns well with the comprehensive needs of the pediatric population [38].

Periodontics

AI has introduced novel possibilities in periodontology, offering tools that improve disease detection, progression monitoring, and risk prediction. Given the multifactorial nature of periodontal disease and its dependence on both clinical and radiographic assessments, AI is especially suited to integrate disparate datasets for more holistic evaluation [39]. The most well-established use of AI in this domain is the automatic detection of alveolar bone loss on radiographs. DL models can analyze intraoral, panoramic, and CBCT images to assess horizontal and vertical bone levels, often with pixel-level precision. These models allow for longitudinal tracking of bone changes, enabling clinicians to monitor disease progression and evaluate treatment outcomes with greater objectivity [40].

In addition to radiographic analysis, AI is being used to classify periodontal status using structured clinical data (e.g., probing depth, clinical attachment loss, bleeding index), combined with demographic and behavioral variables. ML classifiers have been developed to assign patients to different stages or grades of periodontitis based on the updated classification systems. These tools assist in early diagnosis, especially when subtle clinical changes might go unnoticed [41]. AI has also been applied to predict the risk of periodontal disease onset or recurrence, integrating factors such as genetic predisposition, smoking status, systemic conditions (e.g., diabetes), and microbiome profiles. These risk stratification models enable the implementation of personalized preventive protocols, improving long-term outcomes [42]. In surgical periodontology, AI is being explored for flap design optimization and regenerative treatment planning, using imaging data and tissue modeling to assist clinicians in choosing the most appropriate techniques for guided tissue regeneration or bone grafting procedures [43]. Moreover, AI is contributing to plaque detection and oral hygiene assessment. Computer vision tools analyze intraoral photographs or videos to quantify plaque accumulation and highlight areas needing improved hygiene. These technologies are increasingly integrated into patient education apps, enhancing patient engagement and compliance [9].

In essence, AI in periodontology facilitates early diagnosis, personalized risk assessment, and more objective monitoring of treatment success. As models become more robust, they hold the potential to support real-time chairside decisions and patient-specific maintenance strategies [44].

Endodontics

AI plays a growing role in endodontics, where diagnostic accuracy, canal morphology recognition, and outcome prediction are critical. The highly technical nature of root canal therapy makes it an ideal area for the integration of AI to support clinicians in managing anatomical complexity and minimizing procedural errors [45]. One of the most significant applications is in the detection of periapical lesions. DL models, particularly CNNs, have been successfully trained on periapical and panoramic radiographs to identify apical radiolucencies with sensitivity and specificity levels comparable to those of experienced clinicians. These systems are particularly helpful in detecting subtle periapical pathologies that may not be visible in the early stages of disease progression [46].

AI has also been applied to canal detection and segmentation, particularly in teeth with complex canal systems (e.g., mandibular molars or maxillary premolars). Algorithms can interpret CBCT scans and automatically segment root canals, enabling clinicians to visualize canal configurations and plan access cavities with greater precision. This not only improves treatment outcomes but also helps preserve more tooth structure by guiding minimally invasive endodontic access. Furthermore, ML models have been developed to assist in working length determination, analyzing electronic apex locator data and radiographic landmarks to predict accurate canal measurements. Such models reduce reliance on manual interpretation and enhance standardization across practitioners [47]. AI also shows promise in prognostic modeling, where algorithms analyze variables such as preoperative pain, pulp status, root morphology, patient age, and procedural details to estimate the likelihood of treatment success or the need for retreatment. These predictive models are valuable in clinical decision-making and patient counseling, particularly for teeth with a questionable prognosis [48].

Emerging applications include real-time feedback during instrumentation, where AI-integrated systems in endodontic motors may soon help detect canal curvature, file binding, or the risk of ledge formation. Although still in development, such systems may further reduce the incidence of iatrogenic errors. AI in endodontics enhances diagnostic precision, supports anatomically informed treatment planning, and improves prognostic assessment. Its role is expanding from diagnostic support to chairside decision assistance, especially in complex and high-risk cases [49].

Oral Medicine and Oral Pathology

AI has made notable advances in oral medicine and oral pathology, where accurate diagnosis and timely management of mucosal lesions, neoplasms, and systemic oral manifestations are essential. Given the diagnostic complexity in this field, AI offers tools to augment histopathological interpretation, lesion classification, and risk assessment [50]. One of the leading applications is in digital histopathology. AI models trained on digitized microscopic images can classify oral epithelial dysplasia, distinguish between benign and malignant lesions, and identify oral squamous cell carcinoma with high sensitivity and specificity. CNNs are used to detect architectural and cytological abnormalities, reducing the risk of interobserver variability among pathologists and improving diagnostic consistency [51].

In clinical oral examination, AI tools integrated with intraoral photography or smartphone imaging can identify and differentiate lesions such as leukoplakia, erythroplakia, candidiasis, and lichen planus. These tools are particularly valuable in primary care and teledentistry settings where specialist access may be limited. Some mobile applications already use AI to assist nonspecialists in flagging potentially malignant lesions for referral [52]. AI is also being applied to predict malignancy risk using multifactorial models that include lesion appearance, patient demographics, smoking status, and molecular biomarkers. These predictive systems help prioritize cases for biopsy and allow for tailored surveillance protocols [52].

In systemic disease recognition, AI has been explored for detecting oral manifestations of systemic conditions, such as diabetes-related periodontal changes, immunodeficiency-linked ulcerations, and syndromic craniofacial anomalies. By analyzing structured patient data and clinical imaging, AI may assist in interdisciplinary diagnosis and referral [53]. Moreover, NLP-based tools can extract valuable information from pathology reports and clinical notes, enabling automated data mining for research or audit purposes. These tools also support the development of large annotated datasets needed for further training and validation of AI diagnostic models [51]. In essence, AI in oral medicine and pathology enhances diagnostic objectivity, facilitates early detection of serious conditions, and supports the integration of oral health with systemic care. As digital pathology becomes more widespread, AI will play an increasingly central role in the diagnostic workflow of oral lesions and diseases [54]. A consolidated overview of AI applications, benefits, and limitations across major dental specialties is provided in Table 2.

**Table 2:** Summary of artificial intelligence applications

Specialty	Artificial Intelligence Applications	Benefits	Challenges
Orthodontics	Automated cephalometric analysis, treatment monitoring	Efficient planning, remote supervision	Data variability, generalizability
Oral and maxillofacial surgery	Surgical planning, nerve detection, robotic assistance	Improved precision, reduced complications	High cost, limited clinical validation
Restorative dentistry	Caries detection, margin recognition	Early detection, standardization	Diagnostic accuracy in varied clinical settings
Pediatric dentistry	Early caries detection, behavior prediction	Prevention focus, non-invasive diagnostics	Consent and imaging challenges in children
Periodontics	Artificial intelligence-assisted probing, bone loss assessment	Objective measurements, longitudinal monitoring	Limited availability of high-quality training data
Endodontics	Pulp/periapical lesion detection, root canal length prediction	Enhanced diagnostic sensitivity	Image quality dependency, data annotation burden
Oral medicine/pathology	Lesion classification, oral cancer risk prediction	Early intervention, screening potential	Need for histological correlation

Teledentistry and Artificial Intelligence Integration

**Figure 2:**
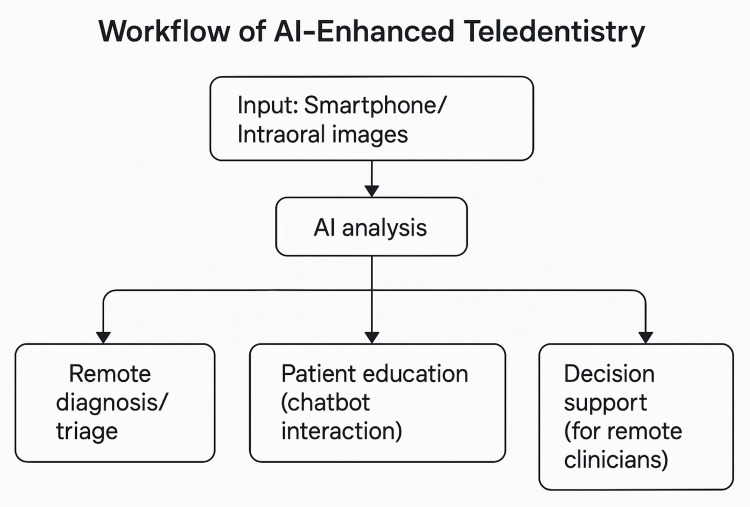
Workflow of artificial intelligence-enhanced teledentistry Image credits: Authors

The fusion of teledentistry and AI is redefining the delivery of oral healthcare by enabling intelligent, accessible, and patient-centered services beyond the confines of conventional clinics [55]. Originally conceptualized to improve care access for underserved populations, teledentistry has matured into a multifunctional ecosystem. Its capabilities are now significantly enhanced by AI, allowing for automated diagnosis, intelligent triage, real-time monitoring, personalized education, and decision support. As digital health tools proliferate in dentistry, the integration of AI into teledentistry platforms serves not only as an adjunct but as a transformative force, expanding the reach, depth, and responsiveness of dental care systems [56]. Figure 2 illustrates the general workflow of AI-enhanced teledentistry, from image acquisition to diagnostic and clinical outcomes.

Artificial Intelligence-Enhanced Diagnostic Support and Remote Triage

One of the most immediate and clinically impactful applications of AI in teledentistry is the automatic analysis of intraoral images. Patients or caregivers can use smartphones or portable intraoral cameras to capture dental photographs or short videos, which are then interpreted by AI algorithms trained on large annotated datasets. These models are capable of detecting caries, gingival inflammation, plaque accumulation, oral mucosal lesions, tooth wear, and orthodontic irregularities. By providing instant feedback and flagging abnormalities for clinician review, AI facilitates an initial triage process in which urgent cases such as dental abscesses or progressive lesions are prioritized for in-person evaluation. This system significantly reduces diagnostic delays, particularly in rural or low-resource communities where dental specialists are scarce. In institutional settings such as nursing homes, schools, or prisons, this approach empowers auxiliary healthcare workers or caregivers to participate in oral health surveillance, supported by AI-generated reports [57].

Remote Monitoring and Longitudinal Follow-Up

Teledentistry platforms augmented by AI enable continuous remote monitoring of dental conditions and treatments. In postoperative care, AI algorithms can assess healing of extraction sockets, flap sites, or implant areas by analyzing wound margins, erythema, and swelling patterns from periodic images. In orthodontics, AI-driven systems analyze tooth position, aligner fit, and interproximal spacing from weekly patient-submitted images, alerting clinicians if an intervention is required or if treatment is progressing suboptimally [58]. For patients undergoing periodontal maintenance, some AI platforms quantify gingival inflammation or plaque accumulation over time and visualize trends to inform scaling frequency or reinforce hygiene education. In pediatric care, parents can upload regular photos to monitor eruption patterns, enamel defects, or oral hygiene in children, particularly useful in early childhood caries surveillance. These features reduce the frequency of unnecessary in-person visits, improve treatment adherence, and empower patients with greater involvement in their own care trajectory [59].

Patient Engagement and Artificial Intelligence​​​​​​​-Driven Oral Health Education

AI-powered chatbots and virtual assistants embedded within teledentistry platforms serve as accessible, interactive sources of patient education. These systems offer step-by-step oral hygiene instruction, dietary advice tailored to caries or erosion risk, explanations of dental procedures in lay language, and reminders for medication use, oral rinses, or follow-up submissions [60]. Using NLP and behavioral data, these AI systems can adapt their messaging style and content to the patient’s age, language, cultural background, and dental knowledge level. For pediatric users, gamified educational content and reward-based feedback loops can promote sustained engagement with oral health practices. For elderly or medically complex patients, AI agents can help caregivers by providing daily hygiene checklists and recognizing patterns suggestive of neglect, dryness, or mucosal disease [61].

Clinical Decision Support and Second Opinions

AI systems in teledentistry can offer real-time clinical decision support to general dentists and remote providers. These systems may suggest potential diagnoses based on image analysis and patient history, provide risk-based stratification such as caries or periodontal status, recommend referral pathways depending on urgency, and assist in material selection or procedural planning [62]. In asynchronous models, in which clinicians review submitted cases after they are uploaded, AI can pre-process and annotate findings, flagging radiographic anomalies, missing data, or suggesting diagnostic codes. In regions without access to specialists, this functionality acts as an AI-generated second opinion, ensuring that less experienced practitioners are still supported by decision frameworks grounded in evidence-based algorithms [63].

Public Health Surveillance and Outreach

From a population-level perspective, AI-enhanced teledentistry contributes to public health surveillance by automatically aggregating data from thousands of remote users [64]. Such systems can map caries and periodontal disease prevalence, identify emerging lesion patterns or geographic clusters of need, monitor oral health inequities among different socioeconomic or ethnic groups, and evaluate the effectiveness of community-based interventions. This provides dental public health authorities with the means to allocate resources more effectively, initiate targeted interventions, and track longitudinal progress toward health equity [65].

Challenges and limitations

Despite its promise, several challenges remain in merging AI with teledentistry. Variability in image quality, particularly in user-submitted photographs affected by motion blur, poor lighting, or inconsistent angulation, can impair AI performance [66]. Many models are trained on datasets lacking demographic diversity, which may reduce diagnostic accuracy in underrepresented populations. Privacy and data security concerns also persist, especially when transmitting and storing patient images through cloud-based platforms that must comply with regulations such as the General Data Protection Regulation (GDPR) or the Health Insurance Portability and Accountability Act (HIPAA). Furthermore, limited digital literacy and internet access in vulnerable populations can hinder the adoption of teledentistry. Additionally, integrating autonomous AI systems into clinical workflows raises regulatory ambiguity concerning accountability, liability, and the legal status of algorithmic decisions [67].

Future outlook

AI-enhanced teledentistry represents a paradigm shift from episodic, clinic-based care to continuous, proactive, and data-driven oral healthcare. As 5G networks, affordable intraoral cameras, and federated AI learning become more accessible, the scalability of such models is expected to expand. Combined with wearable biosensors and real-time salivary diagnostics, future teledentistry platforms may evolve into virtual dental homes capable of comprehensive surveillance, early diagnosis, and preventive intervention across diverse populations [55].

Ethical, Legal, and Regulatory Considerations

While AI is transforming dental care across specialties, its implementation raises profound ethical, legal, and regulatory challenges that must be systematically addressed to ensure safe, fair, and equitable integration. These concerns span patient autonomy, data protection, professional accountability, algorithmic transparency, and the need for global regulatory frameworks. As AI systems shift from decision-support tools to semi-autonomous agents capable of diagnosis and treatment planning, the responsibilities borne by developers, practitioners, and health authorities become increasingly complex [59].

Patient Autonomy, Consent, and Transparency

One of the fundamental ethical principles challenged by AI is informed consent. In traditional dental care, clinicians are expected to explain treatment options, risks, and expected outcomes. With AI systems contributing to clinical decisions, it becomes essential that patients are made aware when an algorithm is involved in their care. However, the complexity and opacity of many AI models, particularly DL systems, make it difficult for both practitioners and patients to fully understand how specific outputs are generated. This raises concerns about whether patients can truly give informed consent when the rationale behind AI-driven decisions is not easily interpretable [68]. Additionally, concerns exist regarding algorithmic bias and fairness. AI models trained on unbalanced datasets may perform worse for underrepresented groups, leading to disparities in diagnosis or treatment. For example, AI tools that interpret radiographs or intraoral photos might have reduced accuracy when applied to populations not well-represented in the training data, such as children, older adults, or individuals with certain ethnic traits. Ensuring algorithmic fairness requires deliberate dataset curation, external validation, and continual performance monitoring across diverse demographic and clinical profiles [69].

Data Privacy, Ownership, and Security

AI systems in dentistry rely heavily on large volumes of patient data, including radiographs, 3D scans, clinical notes, and behavioral data. This raises critical issues surrounding data privacy, particularly as many applications operate via cloud-based platforms and mobile devices. The transmission and storage of sensitive dental and medical data must comply with privacy regulations such as HIPAA in the United States or the GDPR in the European Union. Beyond legal compliance, questions of data ownership remain unsettled [70]. It is not always clear whether patients, clinicians, institutions, or AI developers hold the rights to data used in model training or prediction. In federated learning environments, issues of ownership, consent, and liability become even more complicated. There is also the growing risk of cybersecurity threats, including unauthorized access to AI systems that could manipulate clinical outputs or compromise patient records [71].

Accountability and Professional Responsibility

AI integration challenges traditional notions of professional accountability. When an AI system suggests a diagnosis or treatment plan that results in an adverse outcome, it remains unclear who bears legal and ethical responsibility: the clinician, the software developer, or the institution deploying the technology. In current clinical practice, dentists are ultimately accountable for decisions made in patient care. However, as AI systems evolve and clinicians come to rely on them, the line between support and delegation becomes blurred [72]. Professional guidelines must evolve to clarify how AI tools should be used within the standard of care. Over-reliance on AI recommendations without critical clinical judgment may lead to de-skilling, where clinicians lose the expertise to challenge or verify algorithmic output. Therefore, educational programs must not only train practitioners to use AI tools but also emphasize cognitive oversight, skepticism, and ethical reasoning when interpreting AI-driven recommendations [73].

Regulatory Oversight and Global Standards

The regulatory landscape for AI in dentistry remains underdeveloped and fragmented. In most countries, AI-based tools that assist in diagnosis or treatment are subject to medical device regulations, such as those enforced by the U.S. Food and Drug Administration (FDA) or the European Medicines Agency (EMA). However, these frameworks were not originally designed to assess the dynamic, self-updating nature of ML algorithms, particularly those that evolve with continued data input [74]. There is a pressing need to establish adaptive regulatory models that can assess the safety, validity, and generalizability of AI tools over time. Regulatory bodies must also require transparent documentation of algorithm development, validation cohorts, performance metrics, and limitations. Clear guidelines are needed for labeling AI tools as either assistive or autonomous and for setting standards regarding retraining frequency, post-deployment monitoring, and response to adverse events. Moreover, as AI tools are increasingly developed and deployed across international boundaries, harmonization of global standards becomes essential. Collaborative efforts between dental associations, regulatory agencies, and industry stakeholders are needed to ensure ethical alignment and interoperability of AI systems used in cross-border clinical settings [75].

Addressing Public Trust and Social Acceptance

The long-term success of AI in dentistry depends not only on technical validity but also on public trust. Patients must feel confident that AI is being used to enhance, not replace, human care, and that safeguards are in place to protect their interests. Concerns about depersonalization, data misuse, and “black-box” decision-making may undermine acceptance if not adequately addressed [76]. Transparent communication, patient-centered design, and inclusion of community stakeholders in technology development are key strategies to promote social acceptability. Similarly, dental practitioners must be involved in the evaluation and governance of AI systems to foster a sense of shared responsibility and collaborative oversight [77]. Key opportunities and barriers to integrating AI in dentistry across clinical, educational, and public health domains are outlined in Table 3.

**Table 3:** Opportunities and barriers to artificial intelligence integration in dentistry

Domain	Opportunities	Barriers
Clinical practice	Precision, personalization, efficiency	Trust, clinical validation, cost
Education and training	Simulation, skill assessment, adaptive learning	Resistance to change, curriculum integration
Research	Big data analysis, automated reviews	Data heterogeneity, annotation burden
Public health	Risk mapping, screening programs	Infrastructure limitations, privacy concerns
Ethics and regulation	Enhanced oversight, transparency via audits	Lack of global standards, unclear legal responsibility

Future perspectives and research directions

AI is poised to redefine the landscape of dental care, not only by enhancing clinical workflows but also by promoting more personalized, preventive, and data-driven approaches. While its current applications have shown significant promise, the future of AI in dentistry depends on expanding technical capabilities, fostering interdisciplinary collaboration, and addressing the scientific, ethical, and societal challenges associated with its advancement. The next phase of AI development in dentistry is likely to focus on multimodal data integration, where diverse inputs, such as clinical records, radiographs, intraoral scans, salivary biomarkers, genetic data, and behavioral metrics, are combined to build comprehensive patient profiles. Such holistic models would enable truly personalized treatment planning, with AI systems capable of predicting disease trajectories, identifying optimal intervention strategies, and dynamically adapting care pathways over time. For example, predictive models could forecast the onset of periodontitis or tooth loss years in advance based on genetic susceptibility, microbiome composition, and lifestyle behaviors, enabling preemptive interventions. Another major frontier involves real-time AI applications embedded in clinical devices. Future dental units may incorporate AI-powered sensors capable of monitoring instrument positioning, detecting enamel or dentin boundaries during cavity preparation, and guiding surgical navigation with sub-millimeter precision. These innovations could enhance procedural safety and reduce reliance on intraoperative imaging. In orthodontics, AI-integrated scanners and aligners may continuously track treatment progress, alerting clinicians or patients to deviations and automating minor adjustments through adaptive planning software. A particularly exciting direction is AI-assisted dental robotics. Robotic systems capable of performing restorative, endodontic, or surgical procedures with AI-driven precision are already in early development. These systems have the potential to enhance outcomes, reduce operator fatigue, and support high-accuracy interventions in complex cases. However, widespread adoption will depend on rigorous clinical validation, user interface design, and integration with existing dental workflows. In parallel, AI-enhanced education and training will reshape how dental professionals are prepared for practice. Virtual reality simulators guided by AI can provide personalized feedback on hand skills, decision-making, and diagnostic interpretation, creating a more interactive and self-directed learning environment. Moreover, AI can support continuous professional development by monitoring performance trends, identifying learning gaps, and curating targeted educational content.

**Figure 3:**
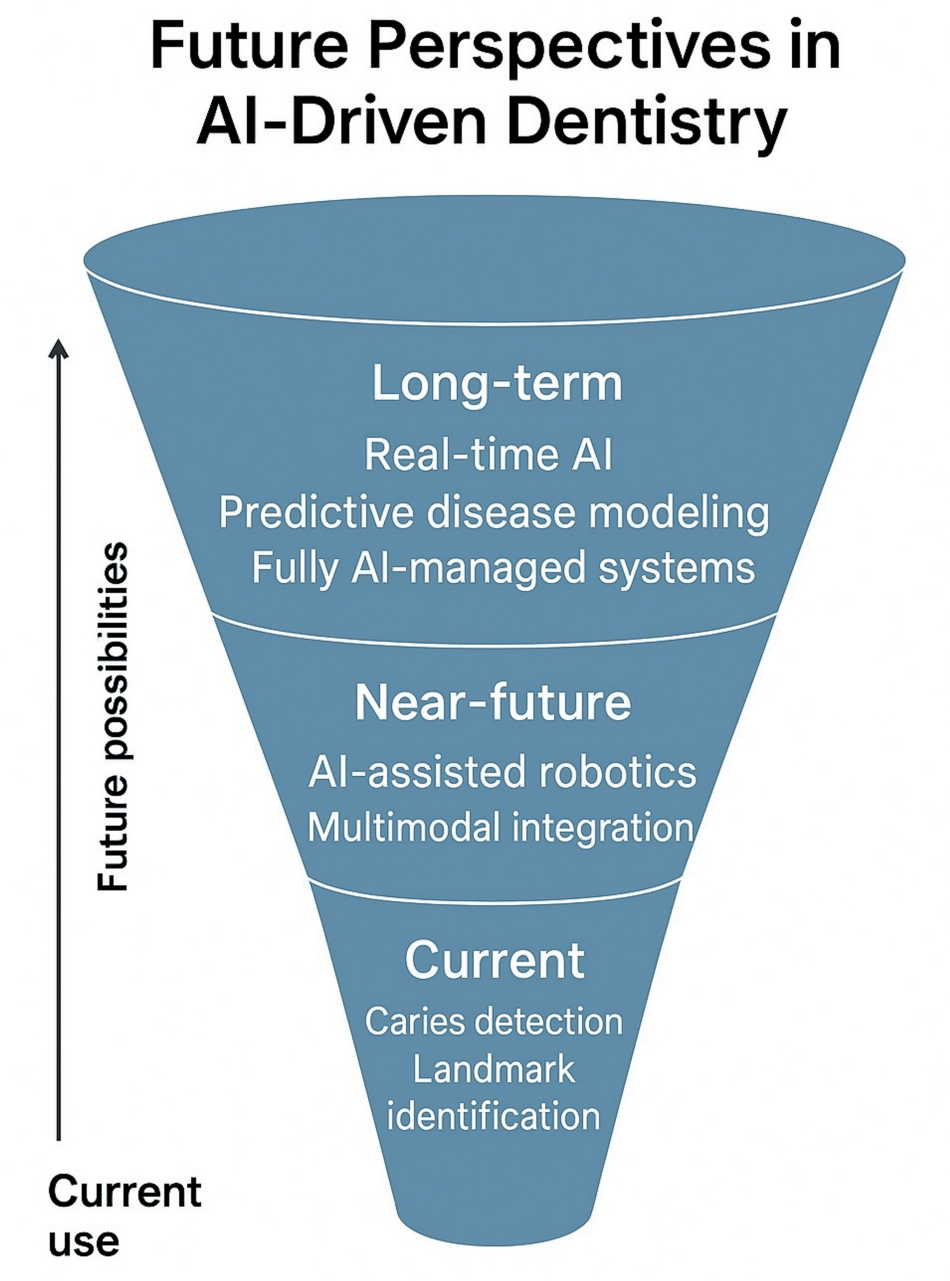
Evolution of artificial intelligence applications in dentistry Image credits: Authors

Despite these promising trajectories, significant research gaps remain. Many current AI models are trained on limited datasets that do not reflect the full diversity of global populations or real-world clinical variation. Future research must prioritize the development of large, high-quality, and ethically sourced datasets that include underrepresented groups, variable imaging conditions, and complex disease presentations. Moreover, longitudinal studies assessing the long-term clinical impact, safety, and cost-effectiveness of AI tools in routine practice are critically needed to inform guidelines and policy. Interdisciplinary collaboration among dentists, computer scientists, data ethicists, and healthcare policymakers will be essential to guide AI innovation in directions that are clinically relevant, ethically sound, and socially responsible. Funding agencies and academic institutions must also play a proactive role in supporting open-access AI research, data-sharing infrastructures, and educational initiatives to build AI literacy among dental professionals. Ultimately, the future of AI in dentistry will not be defined solely by technological sophistication but by its alignment with the values of patient-centered care, equity, and professional integrity. If thoughtfully developed and responsibly deployed, AI has the potential to transform dentistry into a more predictive, precise, and preventive discipline, empowering both clinicians and patients to achieve optimal oral health outcomes (Figure 3).

## Conclusions

AI has become a significant component of contemporary dental practice, providing valuable applications in diagnostics, treatment planning, and patient management. Its integration across all dental specialties highlights its broad clinical utility and transformative potential. While these advancements improve accuracy, efficiency, and accessibility, they also require careful consideration of ethical, legal, and regulatory implications. Ongoing efforts should prioritize the development of diverse training datasets, rigorous clinical validation, and transparent, clinician-guided implementation. When applied responsibly, AI has the potential to advance dentistry toward a more personalized, preventive, and equitable model of care.
